# Student evaluation of the academic advising process in an Iranian medical school

**DOI:** 10.5116/ijme.4f29.a809

**Published:** 2012-02-11

**Authors:** Azra Shamsdin, Mehrnoosh Doroudchi

**Affiliations:** 1 Gastroenterohepatology Research Centre, Shiraz University of Medical Sciences, Iran; 2 Department of Immunology, Shiraz Medical School, Shiraz University of Medical Sciences, Iran

**Keywords:** Academic advising, student evaluation, Iran

## Abstract

**Objective:**

The purpose of this study was to examine student evaluation of the academic advising process in an Iranian medical school.

**Method:**

We conducted a cross sectional survey of all fourth and fifth year students who studied medicine, nursing and laboratory technology. A short version of a validated questionnaire was administrated to 85 students (23 males and 62 females) at Fasa Medical School, Iran.

**Results:**

Of the students, 48(56%) were satisfied with the academic advising process. The descriptive analysis of the study showed that many students (n=72) valued the importance of feedback on student ability in the academic advising process. A further descriptive analysis showed that 34 students (40%) were satisfied that advisers were aware of their records. There was a significant difference between student’s main course (χ^2^_(2)_ = 8.9; p= 0.012) and satisfaction with academic advising. However, the observed differences between female and male students in this study were not statistically significant (χ^2^_(1)_ = 2.2; p= 0.107).

**Conclusions:**

The results of this study reveal a lack of systematic planning, skills and resources for the academic advising process at the Fars Medical School. The results indicate the need for academic staff development initiatives to improve the academic advising process. An ongoing evaluation program of the academic needs of students may help to advisors to provide academic advising and academic support for students in various courses.

## Introduction

Effective academic advising can be a good starting for the best practices in teaching and learning in medicine. In addition, faculty development in the area of academic advising contributes to improve services to students.^1^ However the academic advising process is considerably overlooked in Iranian universities. The purpose of academic education may simply be interpreted as to guide students through the academic life and getting a degree which suites their needs and the society. However, there are more fundamental educational aspects of academic advising that affects their ability of problem solving, reasoning and decision making as well as helping them to feel belonged to the institution and therefore improving the quality of their studies.^2^^,^^3^

Academic advisers are the principle and sometimes unique way by which students are linked to their institution and therefore the quality of their relationship has a great impact on the students’ persistence in their studies.^4^^,^^5^^,^^6^ There are generally two well-known models in academic advising. The first model which is based on an authoritative relationship is known as prescriptive advising. In this approach, which is mostly focused on the student’s course selection, registration and degree requirements; the adviser decides what is the best for the student and prescribes the solutions. ^7^ Despite all the pitfalls of such an approach, even this method can positively impact the performance of students in their studies.^8^^,^^9^ Another approach, which is called developmental advising, is to have the students actively contribute to the decision making process.^7^

In most Iranian universities and colleges, the task of academic advising is performed by the faculty members who are not specifically trained to academically advise students, but they are heavily engaged in the teaching and research in their own specific fields. Paying less attention to the quality of the academic advising process, due to busy schedule and a lack of specific skills of academic advising, may undermined the quality of the academic advising and reduce student satisfaction of the process of education. It has also been shown that by focusing on the needs of students, by continuous improvement of the quality of their experience in the educational institution and by continuous evaluation of the students’ satisfaction, an institution will enter the path to success.^10^

From a cultural perspective, in Iran, women education is not often considered a priority and they usually face more difficulties and obstacles both from their families and the society when they choose to study. Moreover, female students experience more stress in some courses compared to the male students. Taken together, we therefore felt that it is necessary to examine the student perspective on academic advising. To end this, we investigated the following research question: What is student satisfaction with the academic advising process in terms of gender and study subject? Answering this research question may provide useful information for Iranian medical educators in order to improve the quality standards of medical education, which in turn may increase student satisfaction with the academic advising process.

## Methods

### Study participants

We have performed a cross-sectional study of medicine, nursing and laboratory technology students to gain their perspectives of the academic advising process. All fourth and fifth semester students were invited to participate in the study. All 85 fourth and fifth semester students were recruited into our study, consisting of 23 males and 62 females. Table 1 shows the demographic characteristics of students in the sample. Students participated voluntarily without payment in the present study.

**Table 1:** Students demographic characteristics (N=85)

Variable	No	%
Gender		
Male	23	27.1
Female	62	72.9
Missing data	0	0
Total	85	100
Student’s main course		
Medicine	32	37.6
Laboratory	26	30.6
Nursing	27	31.8
Missing data	0	0
Total	85	100

### Instrument

We used an existing questionnaire to collect data. The questionnaire contains 8 Items. It was originally developed and validated by Shiraz University of Medical Sciences as a means of measuring the academic advising process.^11^ Item scores are ranged from 0 to 4(0= no idea; 1= rarely; 2= sometimes; 3= often; 4=always) and students are supposed to choose one statement per item that represents best their satisfaction of academic advising. To reduce missing data in the items, we modified the questionnaire, consisting of 7 items, which measure student perspectives of the academic advising process at a single point in time. Each item on the questionnaire was rated in one of four groups, namely, no idea, satisfied, partially satisfied and unsatisfied. For the purpose of this study, selecting partially satisfactory or satisfactory indicates that students are actually satisfied with their academic advising.

### Procedures

The study was approved by the institution review board at Fasa Medical School, Iran. The study was conducted between October 2009 and October 2010 at the Fasa Medical School, Iran. The questionnaire was completed anonymously and only group data would be reported. Students were invited to complete the questionnaire after the end of the microbiology lecture. Before students began completing the questionnaire, students were fully informed about the purpose of the study in which they were participating. The questionnaire took approximately 5 to 7 minutes to complete. This is a considerable reduction in time commitment for students. We explained the students that participation is voluntary and that they can decline to participate at any time without penalty and that refusal to participate would not affect their course.

### Statistical analysis

Data were analysed using SPSS version 11.5. The descriptive and inferential analyses were used to gain the relationship between the independents and dependents variables. The chi-square test was used to determine the relationship between independent variable (gender and students with various courses) and satisfaction with academic advising (dependent variable).

## Results

A total of 85 students participated in the study. Of them, 23 (27.1%) and 62 (72.9%) were male and female, respectively. Thirty-two medical students (37.6%), 27 (31.8%) nursing students and 26 (30.6%) laboratory technology students completed the survey. Females made up the majority of the study participants students, 17 in medicine, 25 in nursing and 20 in laboratory technology. The value of Cronbach’s alpha was 0.95, indicating a satisfactory reliability.

The results indicated that 48(56%) of the students were somewhat satisfied with their overall academic advising at the Fasa Medical School. The vast majority of students (n=72, with 85% satisfaction) reported that they need to advise on their own abilities and potentials. Some students were satisfied with the coordination of learning experiences through course and career planning and academic progress review (n=43, 50% satisfaction) followed by frequent assessment and review of students’ files and records.

**Figure 1:**
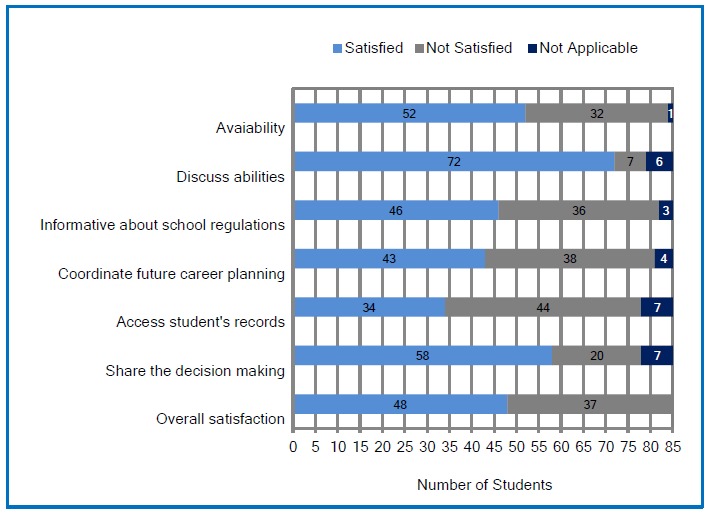
Student satisfaction with academic advising on each item (N=85)

Figure 1 shows the percentage of satisfaction on each item. It is apparent from this figure that the students were willing to be advised about their own abilities and potentials and shared their decision to improve learning experiences. Some students (37.7%) reported that their advisers were not consistently available for the consultation and they had difficulty to provide essential information about the school and to assist students on courses and career planning. The two latter qualities refer to the appropriate training of the advisor for the task.

Further analyses showed the association between student satisfaction with academic advising and gender and students courses. As shown in Table 2, although male students had a higher level of satisfaction with the academic advising process than did female students, the difference was not statistically significant (χ^2^_(1)_ = 2.2; p = 0.107). Referring to Table 2, there was a significant difference between student course and satisfaction with academic advising (χ^2^_(2)_ = 8.9; p= 0.012). As shown in Table 2, medical students were more satisfied with academic advising in comparison with other students. A further analysis also showed that the highest satisfaction was in male medical students and the lowest satisfaction in female nursing students. More male medical students (12 out of 15 male) than female (12 out of 17 female) were satisfied with academic advising. More male laboratory technology students (4 out of 6) than female (11 out of 20) were satisfied with academic advising. In comparison with other courses, the satisfaction among male and female nursing student was low.

**Table 2:** Students satisfaction with academic advising according to gender and student’s main course (N=85)*

Variable	Satisfaction (%)	Non-satisfaction (%)	Total (%)
Gender^†^			
Female	32(51.6)	30(48.4)	62(100)
Male	16(69.6)	7(30.4)	23(100)
Total	48(56.5)	38(43.5)	85(100)
Student’s main course^‡^			
Medicine	24(75)	8(25)	32(100)
Laboratory	15(57.7)	11(42.3)	26(100)
Nursing	10(37)	17(63)	27(100)
Total	49(57.7)	36(42.3)	85(100)

*There was no missing data in terms of gender and student’s main course; ^†^χ^2^_(1)_ = 2.2; p= 0.107; ^‡^χ^2^_(2)_ = 8.9; p= 0.012

## Discussion

The purpose of this study was to examine student evaluation of the academic advising process in an Iranian medical School. Our study showed that, in different study courses, Fasa student’s satisfaction with academic advising is low. In addition, it appears to the academic advising procedures is a mix of prescriptive and developmental approaches in Fasa Medical School. The results of the study showed that the advisers had enough motivation to involve in a one-to-one relationship with the students and also were willing to discuss and share the decision making process with them. However, these approaches do not seem to be systematically conducted as the advisers failed to provide evidence of training and awareness of their own approaches. Not being consistently available and a lack of regular assessment of students’ files and records could be due to the fact that the academic advisers are mostly preferred to involve in teaching and learning students and less pay attention to the issues of academic advising of students. This may be attributed to the fact that the pathway of promotion for advisers is teaching and research rather than advising students.^12^

The lack of ability to create learning experience through courses, career planning and academic progress and failing to provide useful information about school regulations and resources may be an indication of a lack of systematic planning for the academic advising procedure. It may be argued that a lack of skills and resources resulted in an uncoordinated academic advising procedure that was not planned appropriately. It has been well documented that advisors require possessing specific skills to provide useful information for students; in particular the skills of communication, questioning and referral are essential.^13^ Our results also show that students were not satisfied with their advising at the referral facilities provided by the advisers in Fasa Medical School. The referral skill requires a comprehensive knowledge of the campus life, regulations and opportunities available for the students. Such knowledge, however, on the one hand depends on the availability of resources and information and on the other hand depends on the efforts made by the advisers.^14^ Furthermore, in Iran, the cultural issues may influence the student-adviser communication, which in turn can influence the advisor’s style of relying academic advice, particularly female students. Our study shows that female students were not satisfied with their academic advising in comparison with male students. It may be argued that the cultural aspects of communication have influenced the quality of advice provided for female students. The results of this study revealed that male medical students were more satisfied than the other students with respect to academic advising, while the lowest satisfaction rate was reported among nursing female students. In our study, nursing students were advised by nursing academic staff, while laboratory technology and medical students were advised by basic science academic staff. This study does not show whether basic science academic staff can provide better academic advising for students, where two parties do not have the same main subject. This suggests, however, more research on this topic needs to be undertaken before the association between student’s main subjects and academic adviser’s main subject is more clearly understood.

Higher satisfaction among medical students compared to other students may indicate that advisors provide students with good information to improve their learning process, or students were more inclined to communicate with their own advisers to improve.

### Limitations of the study

This study has some limitations that warrant attention. Therefore the results of this study should be interpreted cautiously. First, this study was conducted at a single medical school, therefore cannot be generalized to the population. Second, the study relies merely on a self-administered questionnaire of academic advising. It could be argued that students might be reflected a socially desirable response and not actual experiences. This may suggest other data collection procedures to further research in this area. Finally, we conducted a cross-sectional study with more female students in the sample. Therefore, it is possible that the cohort effects explain the lack of difference among female and male students. Longitudinal studies to track changes over a period of time are therefore suggested. Some qualitative studies can be used as complementary strategies.

## Conclusions

The relationship between advisors and advisees should be optimised to fulfil the academic needs of students, which in turn increases their satisfaction with the academic advising process. Our results suggest that Fasa Medical School should be engaged in development of the academic advising process. This should be based on the academic needs and potential demands of students. Moreover, a systematic and periodic evaluation of freshmen questions and needs along with the solutions can be used to establish a database of information as a resource for the academic advisers. As academic advising is an important key in students' development, satisfaction, academic success, recruitment, and retention, developing valid and reliable instruments for assessing academic advising is essential.^15^ In addition, an on-going evaluation program of the academic needs of students may be useful for advisors to provide academic advising and academic support for students in various courses. More importantly, continuous and specific training of advisors is necessary for them to be able to provide satisfactory advice for students.

## 

### Conflict of Interest

The authors declare that they have no conflict of interest.

## References

[1] Harrison E. Faculty perceptions of academic advising: "I don't get no respect". Nursing Education Perspectives. 2009;30(4):229-33 19753856

[2] Crookston BB. A developmental view of academic advising as teaching. Journal of National Association of College Admissions Counselors. 1994;14(2):5-9

[3] Facione PA Sánchez CA Facione NC Gainen J. The disposition toward critical thinking. The Journal of General Education. 1995;44(1):1-25

[4] Hale MD Graham DL. Johnson DM. Are students more satisfied with academic advising when there is congruence between current and preferred advising styles? College Student Journal. 2009;43(2):313-324

[5] Noel LT. College student retention-A campus-wide responsibility. Journal of National Association of College Admissions Counselors. 1976;21:33-36

[6] Glennen RE Farren PJ Vowell FN. How advising and retention of students improves fiscal ability. Journal of National Association of College Admissions Counselors. 1996;16:38-41

[7] Crookston BB. A developmental view of academic advising as teaching. Journal of College Student Personne. 1972;13:12-17

[8] Seidman A. The evaluation of pre/post admission/counseling process at a suburban community college: impact on student satisfaction with the faculty and the institution, retention and academic performance. College and University. 1991;66(4):223-32

[9] Elliot K Healy M., Key factors influencing student satisfaction related to recruitment and retention. Journal of Marketing for Higher Education. 2001;10(4):1-11

[10] Low L. Are college students satisfied? A national analysis of changing expectations. New Agenda Series. USA Group: Indianapolis, IN; 2000.

[11] Shiraz University of Medical Sciences. Student evaluation of the academic advising process. Shiraz 2011 [cited 22 July 2009]; Available from: http://education.sums.ac.ir/icarusplus/export/sites/education/rules/x_x_x_x.doc

[12] Shiraz University of Medical Sciences. Executive bylaw of letter no. 214116. Shiraz: Shiraz University of Medical Sciences; 2010.

[13] Gasper ML. Building a community with your advisees. Nurse Educator. 2009;34(2): 88-94 20339340 10.1097/NNE.0b013e3181990eb9

[14] Frost SH, Academic advising for student success. A system of shared responsibility [ASHE-ERIC higher education report no. 3] Washington DC: The George Washington University, School of Education and Human development; 1991.

[15] Harrison E. Development and pilot testing of the faculty advisor evaluation questionnaire. Journal of Nursing Education. 2011;30:1-5 10.3928/01484834-20111230-04 22201275

